# Transcriptomics in Tumor and Normal Lung Tissues Identify Patients With Early-Stage Non–Small-Cell Lung Cancer With High Risk of Postsurgery Recurrence Who May Benefit From Adjuvant Therapies

**DOI:** 10.1200/PO.22.00072

**Published:** 2022-09-15

**Authors:** Vladimir Lazar, Nicolas Girard, Eric Raymond, Jean-François Martini, Susan Galbraith, Jacques Raynaud, Catherine Bresson, Benjamin Solomon, Shai Magidi, Hovav Nechushtan, Amir Onn, Raanan Berger, Haiquan Chen, Amal Al-Omari, Sadakatsu Ikeda, Ulrik Lassen, Marina Sekacheva, Enriqueta Felip, Josep Tabernero, Gerald Batist, Alan Spatz, C.S. Pramesh, Philippe Girard, Jean-Yves Blay, Thierry Philip, Ioana Berindan-Neagoe, Angel Porgador, Eitan Rubin, Razelle Kurzrock, Richard L. Schilsky

**Affiliations:** ^1^ Worldwide Innovative Network—WIN Consortium, Villejuif, France; ^2^ Institut Curie, Paris, France; ^3^ Institut du Thorax Curie—Institut Montsouris, Paris, France; ^4^ Groupe Hospitalier Saint-Joseph, Paris, France; ^5^ Pfizer Inc, San Diego, CA; ^6^ AstraZeneca Plc, Cambridge, United Kingdom; ^7^ Avera Cancer Institute, Sioux Falls, SD; ^8^ Hadassah Sharett Institute of Oncology, Jerusalem, Israel; ^9^ Sheba Medical Center, Tel-Hashomer, Israel; ^10^ Fudan University Shanghai Cancer Center, Shanghai, China; ^11^ King Hussein Cancer Center, Amman, Jordan; ^12^ Tokyo Medical and Dental University, Tokyo, Japan; ^13^ Rigshospitalet, Copenhagen, Denmark; ^14^ Sechenov First State Medical University, Moscow, Russia; ^15^ Vall d'Hebron Hospital Campus and Institute of Oncology (VHIO), UVic-UCC, Barcelona, Spain; ^16^ Segal Cancer Center, Jewish General Hospital, McGill University, Montréal, Canada; ^17^ Tata Memorial Hospital, Tata Memorial Center, Homi Bhabha National Institute, Mumbai, India; ^18^ Institut Mutualiste Montsouris, Paris, France; ^19^ Center Leon-Bérard, Lyon, France; ^20^ Unicancer, Paris, France; ^21^ Iuliu Hatieganu University of Medicine and Pharmacy, Cluj-Napoca, Romania; ^22^ Ben-Gurion University of the Negev, Be'er Sheva, Israel

## Abstract

The prognosis of patients with non–small-cell lung cancer (NSCLC), traditionally determined by anatomic histology and TNM staging, neglects the biological features of the tumor that may be important in determining patient outcome and guiding therapeutic interventions. Identifying patients with NSCLC at increased risk of recurrence after curative-intent surgery remains an important unmet need so that known effective adjuvant treatments can be offered to those at highest risk of recurrence.

**METHODS:**

Relative gene expression level in the primary tumor and normal bronchial tissues was used to retrospectively assess their association with disease-free survival (DFS) in a cohort of 120 patients with NSCLC who underwent curative-intent surgery.

**RESULTS:**

Low versus high Digital Display Precision Predictor (DDPP) score (a measure of relative gene expression) was significantly associated with shorter DFS (highest recurrence risk; *P* = .006) in all patients and in patients with TNM stages 1-2 (*P* = .00051; n = 83). For patients with stages 1-2 and low DDPP score (n = 29), adjuvant chemotherapy was associated with improved DFS (*P* = .0041). High co-overexpression of *CTLA-4*, *PD-L1*, and *ICOS* in normal lung (28 of 120 patients) was also significantly associated with decreased DFS (*P* = .0013), suggesting an immune tolerance to tumor neoantigens in some patients. Patients with DDPP low and immunotolerant normal tissue had the shortest DFS (*P* = 2.12E–11).

**CONCLUSION:**

TNM stage, DDPP score, and immune competence status of normal lung are independent prognostic factors in multivariate analysis. Our findings open new avenues for prospective prognostic assessment and treatment assignment on the basis of transcriptomic profiling of tumor and normal lung tissue in patients with NSCLC.

## INTRODUCTION

Lung cancers are classified into two major subtypes, small-cell lung cancer and non–small-cell lung cancer (NSCLC), with the latter accounting for approximately 80% of all primary lung cancers.^1^ NSCLC, a leading cause of cancer deaths, represents a heterogeneous group of neoplasms, mostly comprising squamous cell carcinoma (SCC), adenocarcinoma (AC), and large-cell carcinoma (LCC).^2^

**CONTEXT**

**Key Objective**
Understanding the biological features of both the tumor and the host normal lung tissues that identify patients with non–small-cell lung cancer at increased risk of recurrence after curative-intent surgery who may benefit of adjuvant therapies.
**Knowledge Generated**
We identified key tumor-specific transcribed genes associated with the postsurgery disease-free survival (on the basis of differential mRNA expression between tumor and normal tissues obtained from patients with resected non–small-cell lung cancer). We also examined transcriptomic biomarkers in resected normal lung tissues that reflected the immune system status of the host and identified an immune-tolerant profile associated with a high risk of tumor recurrence.
**Relevance**
An immune-tolerant profile is associated with a shorter disease-free survival after curative-intent surgery because of a limited immune response to disseminated tumor cells and opens new therapeutic avenues.

The histology and TNM staging system on the basis of tumor size, nodal involvement, and the presence of distant metastases are the current standard for prognostication.^3-6^ At diagnosis, there is a strong correlation between the tumor stage and survival. Patients with stage 1 tumors at diagnosis are usually cured by surgery alone, with a 5-year survival rate of 90%. The 5-year survival rate of patients with stage 2 tumors drops to 44%.^4,5^ Adjuvant chemotherapy is recommended for patients considered at high risk of recurrence (commonly stage 2 and 3A), even if only 5%-7% of such patients actually benefit.^7-9^ It is thus critical to identify those patients with early-stage disease at high risk of recurrence after surgery who might have the greatest potential to benefit from postoperative adjuvant therapy.

Despite the considerable number of studies reporting biological characteristics correlating with outcomes, such as *TP53*, *EGFR*, and *RAS* mutations^10^ as well as gene expression-based markers for lung cancer,^11^ there is still no consensus on prognostic and predictive molecular signatures that are useful in the clinic^12^ related in part to high interpatient basal gene expression variability that confounds interpretation of tumor gene expression profiling.^13,14^ Meanwhile, immune checkpoint inhibitors are now demonstrating efficacy in the perioperative setting, stressing the need for a better stratification of patients.^15-17^ Our hypothesis is that the risk of recurrence after surgery can be identified by differences in gene expression between tumor and normal lung tissue and expression of immune genes in the normal lung tissue. We, therefore, performed a novel transcriptomic analysis of matched tumor and normal lung tissues from patients with resected NSCLC^18^ that enables control of interpatient variability of the basal level of gene expression.

## METHODS

### Study Rationale Design

We hypothesized that two key biological factors could explain the risk of recurrence of patients with early-stage NSCLC after curative-intent surgery: (1) differential gene expression between tumor and normal lung tissues from the same patient that indicate activated molecular pathways, using the Digital Display Precision Predictor (DDPP) method^19^ and (2) immune-competent versus immune-tolerant status of the host assessed by the level of activation of the immune regulatory genes programmed death-ligand 1 (PD-L1) and cytotoxic T-cell lymphocyte (CTLA)-4 in normal lung that may limit the immune response to tumor cells.^20^ An immune-competent profile would enable elimination of circulating tumor cells released during surgery and of established micrometastases by activated immune cells.^21,22^ Conversely, activated immune checkpoints PD-L1 and CTLA-4 may dampen the antitumor responses triggered by the T cells that recognize tumor neoantigens.

#### Patients and tissue samples.

This in-silico study used data generated and published by the European Union-funded (FP6) Integrated Project CHEMORES.^18^ CHEMORES was an observational study that followed 123 early-stage patients with NSCLC who underwent surgery at the Institut Mutualiste Montsouris between January 2002 and June 2006. Patients were treated with surgery alone (n = 62) or surgery followed by adjuvant chemotherapy (n = 61) and were followed for up to 92 months after surgery, when the last patient relapsed. Tumor and normal lung tissues obtained from surgical resection were handled according to the Tumor Analysis Best Practices Working Group.^23^ DNA sequencing, differential gene expression between tumor and normal fresh frozen tissues, and outcomes were available. A full description of the genomic investigation is available in Ref. 18.

#### Ethics.

The biobanking study was approved by the Institut Mutualiste Montsouris's Ethics Committee.

#### Computational and statistical analyses.

The statistical analysis was conducted using Knime (Konstanz Information Miner), a graphical environment for data analysis pipeline development using R code snippets as required.^24^ The complete Knime workflow is available upon request. Multiple comparison correction was performed on the *P* values using false discovery rate^25^, and genes with a false discovery rate < 0.05 were considered significant. Patients were classified on the basis of the DDPP score^19^ or expression levels (high *v* low) of particular immune checkpoint genes using k-mean clustering (k = 2). Survival analysis was carried out in R on the 120 patients with NSCLC who had full clinical data available using univariate and multivariate Cox regression models (packages survival and survminer).^26,27^

#### DDPP algorithm.

DDPP is a novel tool that relates the differential expression of genes in tumor versus normal tissue to clinical outcome of patients with cancer of any histology.^19^ The full transcriptome (approximately 19,500 genes) was analyzed for tumor and normal bronchial tissue of each patient and correlated (Pearson correlation) with the disease-free survival (DFS). The most significant set of genes (on the basis of *P* value and correlation coefficient) were selected as the correlator for each histology and chemotherapy group: AC_no-chemotherapy (NC), AC_chemotherapy, SCC_NC, SCC_chemotherapy, LCC_NC, and LCC_chemotherapy resulting in six different linear regression correlators, one for each group. Detailed methodology is presented in the Data Supplement.^19^

### Expression of Immune Checkpoint Genes in Normal Lung Tissues

To investigate the contribution of host immune status to risk of recurrence, we examined expression of various immune checkpoint genes in normal lung tissue from these patients. For each gene, we classified each patient as high or low on the basis of the expression level in the normal tissue. *PD-L1*, *CTLA-4*, and *ICOS* showed the most significant variability (by Student's *t* test): *P* = 5.61E–27, *P* = 2.1E–26, and *P* = 2.78E–18, respectively, and were retained for correlations with clinical outcome.

## RESULTS

Transcriptomic data on 123 patients with NSCLC were available, and full clinical data were available for 120 patients. The median age of patients in the CHEMORES study was 63 years (range, 41-85 years); 89 patients (72%) were men. The histologic subtypes of tumor were AC (n = 57), SCC (n = 50), and LCC (n = 16), as shown in Table 1. TNM distribution was stage 1—56 patients (46%); stage 2—27 (22%); stage 3—32 (26%); stage 4—5 (4%), and undetermined—3 (2%). The patients with stage 4 were clinical stage 3 before surgery but were upstaged on the basis of surgical findings (Table 1).

**TABLE 1.**
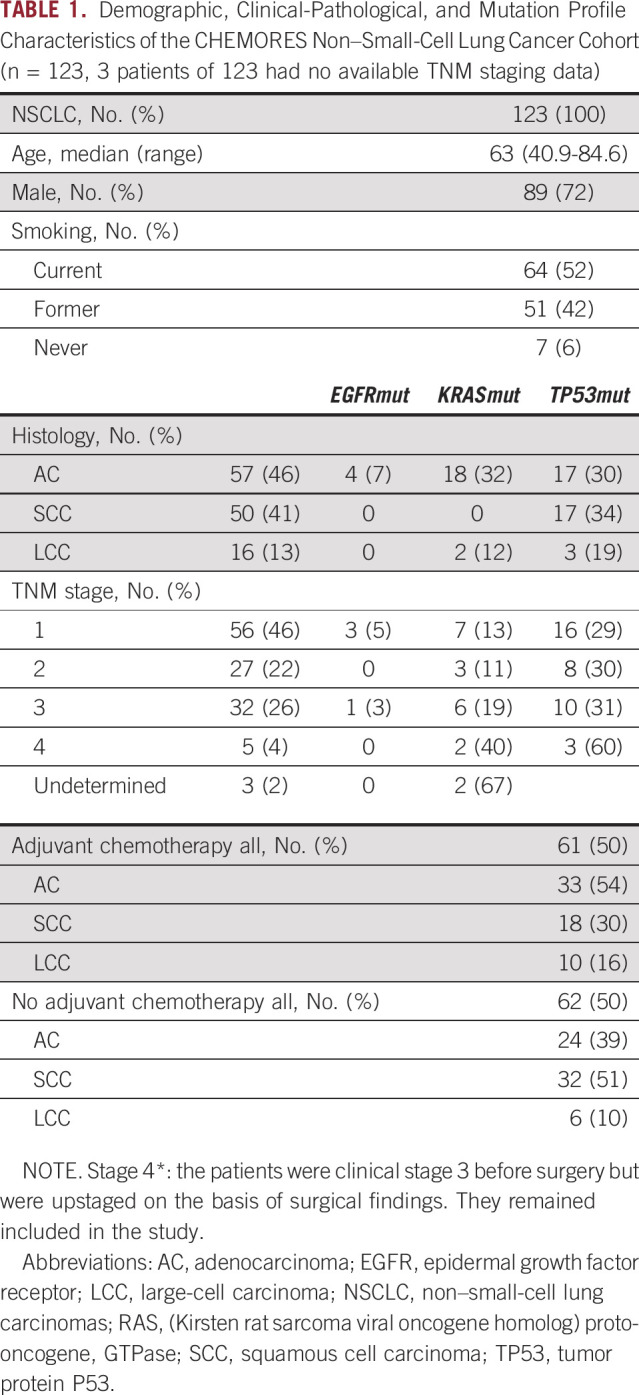
Demographic, Clinical-Pathological, and Mutation Profile Characteristics of the CHEMORES Non–Small-Cell Lung Cancer Cohort (n = 123, 3 patients of 123 had no available TNM staging data)

At the discretion of the treating oncologist, adjuvant platinum-based chemotherapy was administered to 61 patients, of whom 33 had AC (54%), 18 SCC (30%), and 10 LCC (16%). The remaining 62 patients were treated with surgery only, of whom 24 had AC (39%), 32 SCC (51%), and six LCC (10%). DFS after surgery ranged from 3 to 92 months.

### Identification of Tumor Biomarkers Associated With Outcome Using DDPP

**FIG 1.**
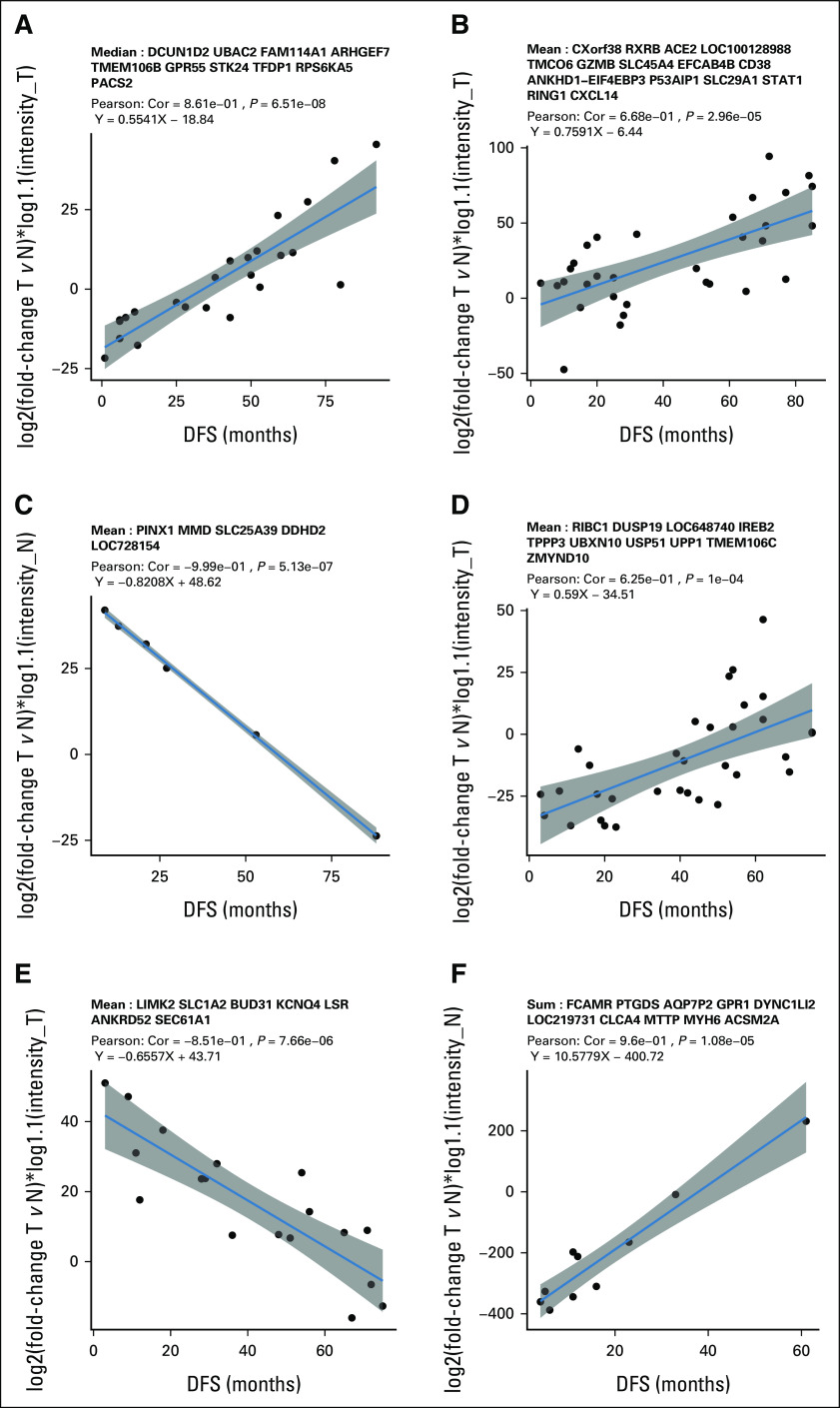
Identification of DDPP predictors of the DFS for patients with non–small-cell lung cancer. Pearson correlation plots of the DDPP predictors with the DFS (in months) presented on *x*-axis. (A) 10-gene predictor of DFS for 24 patients with AC treated with curative surgery only; *y*-axis: median value of log2-based fold-changes tumor versus normal multiplied by log1.1 on the basis of the intensities in tumor values for each of the genes selected; (B) 15-gene predictor of DFS for 32 patients with SCC treated with surgery only; *y*-axis: mean value of log2-based fold-changes tumor versus normal multiplied by log1.1 on the basis of the intensities in tumor values for each of the genes selected; (C) 5-gene predictor of DFS for six patients with LCC treated with curative surgery only; *y*-axis: mean value of log2-based fold-changes tumor versus normal multiplied by log1.1 on the basis of the intensities in normal values for each of the genes selected; (D) 10-gene predictor of DFS for 33 patients with AC treated with curative surgery and adjuvant chemotherapy; *y*-axis: mean value of log2-based fold-changes tumor versus normal multiplied by log1.1 on the basis of the intensities in tumor values for each of the genes selected; (E) 7-gene predictor for 18 patients with SCC treated with curative surgery and adjuvant chemotherapy; *y*-axis: mean value of log2-based fold-changes tumor versus normal multiplied by log1.1 on the basis of the intensities in tumor values for each of the genes selected; (F) 10-gene predictor of DFS for 10 patients with LCC treated with curative surgery and adjuvant chemotherapy; *y*-axis: sum value of log2-based fold-changes tumor versus normal multiplied by log1.1 on the basis of the intensities in tumor values for each of the genes selected. AC, adenocarcinoma; Cor, correlation; DDPP, Digital Display Precision Predictor; DFS, disease-free survival; LCC, large-cell carcinoma; N, normal; PFS, progression-free survival; SCC, squamous-cell carcinoma; T, tumor.

We analyzed the whole transcriptome by correlating for each gene the differential expression in tumor versus normal lung with the DFS observed in each patient treated by surgery only or by surgery followed by adjuvant chemotherapy and identified six specific gene expression signatures, one per histology and treatment group. The detailed methodology on the basis of DDPP is presented in the Data Supplement. Figure 1 shows the optimal predictor identified for each histology and treatment group, the composition of genes of each specific predictor, and the correlations with DFS (*P* value and correlation coefficients as well as the linear equations linking gene expression to outcome).

The cutoff for low versus high DDPP was determined by k-means clustering (k = 2), as described in the Data Supplement; 75 patients were classified as DDPP high and 45 patients as DDPP low while the DDPP threshold was identified as approximately –5.7 months (the DDPP-high group was ranged from –5.61 to 94.36 while the DDPP-low group was ranged from –47.44 to –5.86).

**FIG 2.**
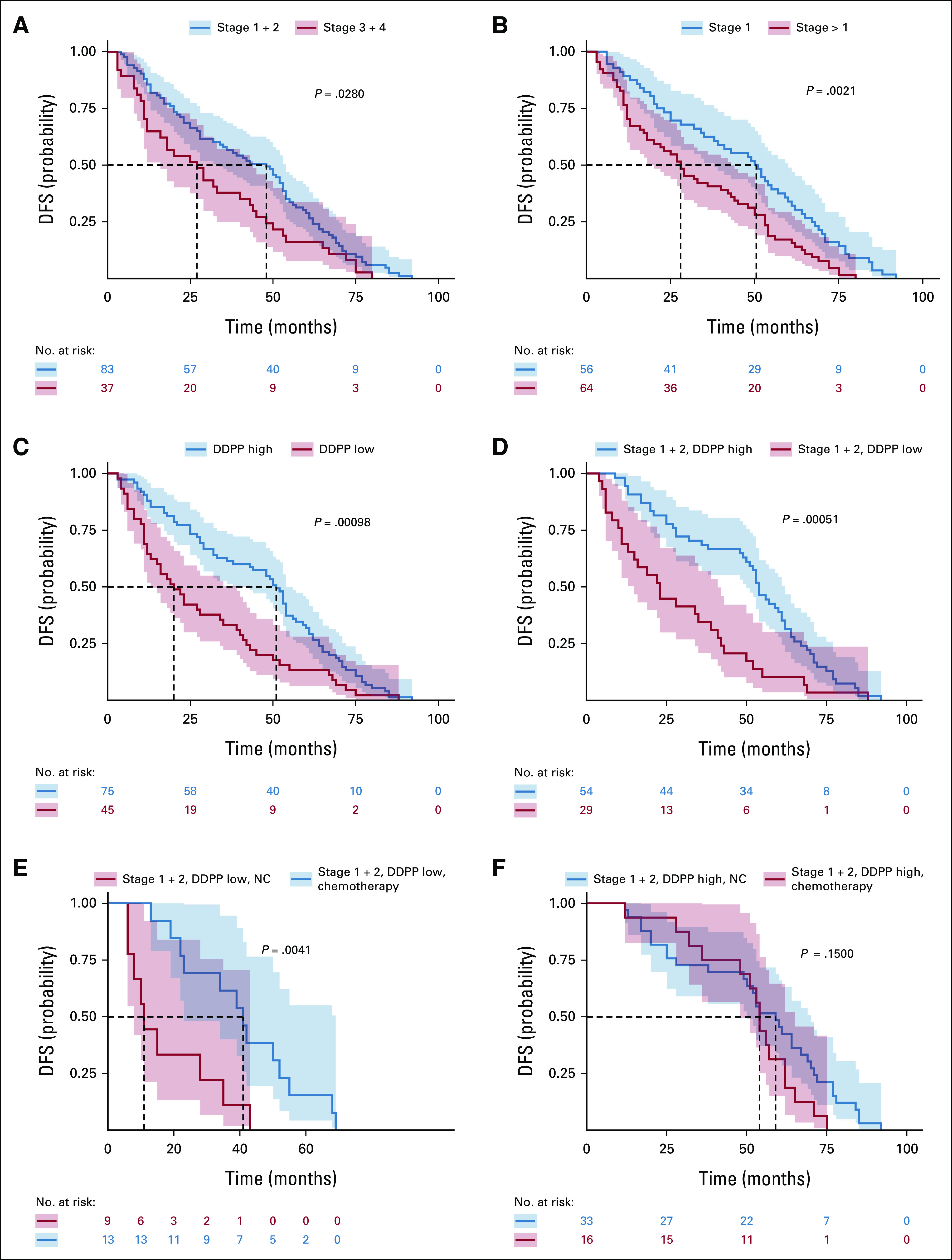
Stage and DDPP score Kaplan-Meier DFS probability plots. The DFS probability of patients: (A) diagnosed at stages 1 + 2 (blue line, median 48 months) and stages 3 + 4 (red line, median 27 months; *P* = .028 with a 95% CI); (B) diagnosed at stage 1 (blue line, median 50.5 months) versus stages > 1 (stages 2 + 3 + 4; red line, median 28 months; *P* = .0021); (C) indicated with high DDPP values (blue line, median 51 months) or with low DDPP values (red line, median 20 months; *P* = .00098); (D) diagnosed with stages 1 + 2 and indicated with high DDPP values (blue line, median 54 months) and diagnosed with stage 1 + 2 and indicated with DDPP low values (red line, 29 months; *P* = .00051); (E) diagnosed with stages 1 + 2 and indicated with low DDPP values who were not treated with adjuvant chemotherapy (NC, blue line, median 11 months) and treated with adjuvant chemotherapy (chemotherapy, red line, median 41 months; *P* = .0041); and (F) diagnosed with stages 1 + 2 and indicated with high DDPP values who were not treated with adjuvant chemotherapy (NC, blue line, median 59 months) and treated with adjuvant chemotherapy (chemotherapy, red line, median 54 months; *P* = .15 with a 95% CI). The table shown below the curves indicates the number of patients from each group who survived up to a given time (according to the values displayed in the *x*-axis in each figure) during the follow-up period (until 92 months when the recurrence of the last patient occured). Dotted lines point the median DFS in each caption. DDPP, Digital Display Precision Predictor; DFS, disease-free survival; NC, no-chemotherapy.

The Kaplan-Meier (KM) analysis was used to visualize the impact of TNM staging and the DDPP level (high and low) on DFS. Figures 2A and 2B show that patients with low TNM stages have significantly better DFS versus patients with high TNM stages. Figure 2C demonstrates that DDPP-low patients have a shorter DFS probability than DDPP-high patients (*P* = .00098). We further investigated the combination of TNM stage and DDPP score. When the analysis is restricted to TNM stages 1 + 2 only, DDPP-low patients have shorter DFS when compared with DDPP-high patients (*P* = .00051, log-rank test; Fig 2D). Importantly, stage 1 + 2, DDPP-low patients who received postoperative adjuvant chemotherapy had a longer DFS than those who did not (*P* = .0041, log-rank test; Fig 2E). In contrast, stage 1 + 2 DDPP-high patients did not benefit from postoperative adjuvant chemotherapy (*P* = .15, log-rank test; Fig 2F).

#### Analytical validation of the findings.

##### Specificity.

To assess the robustness and specificity of the DDPP predictors, we performed random selections of 10 genes across the whole transcriptome and correlated their relative expression with DFS of the 24 patients with AC treated with curative surgery only, repeating this analysis 100,000 times. None of the random combinations of genes correlated significantly with DFS.

##### Replicates.

We performed bootstrap analyses on patients with AC treated with curative surgery only (n = 24) to assess the stability of the biomarker signature, reiterating 100,000 resampling combinatorial analyses. At each reiteration, 18 patients were selected randomly to constitute a training set and were used to establish the correlator following the DDPP algorithm methodology. The correlator was then used to predict the DFS of the six patients left out. Reiterative experiments showed a high stability of the predictor: In 75% of the reiterations, the correlator was stable and identical with the correlator obtained on the entire AC cohort. In the remaining 25%, there were slight variations in the number of genes (seven to nine genes) retained in the predictor. Furthermore, a significant correlation was observed between the predicted and observed progression-free survival (R = 0.75, *P* = 2E–26; Data Supplement).

Applying the methodology for each histology group without separating into treatment groups (chemotherapy versus nonchemotherapy) resulted in a poor predictor of DFS.

### Expression of Immune Checkpoint Genes in Normal Lung Tissues

**FIG 3.**
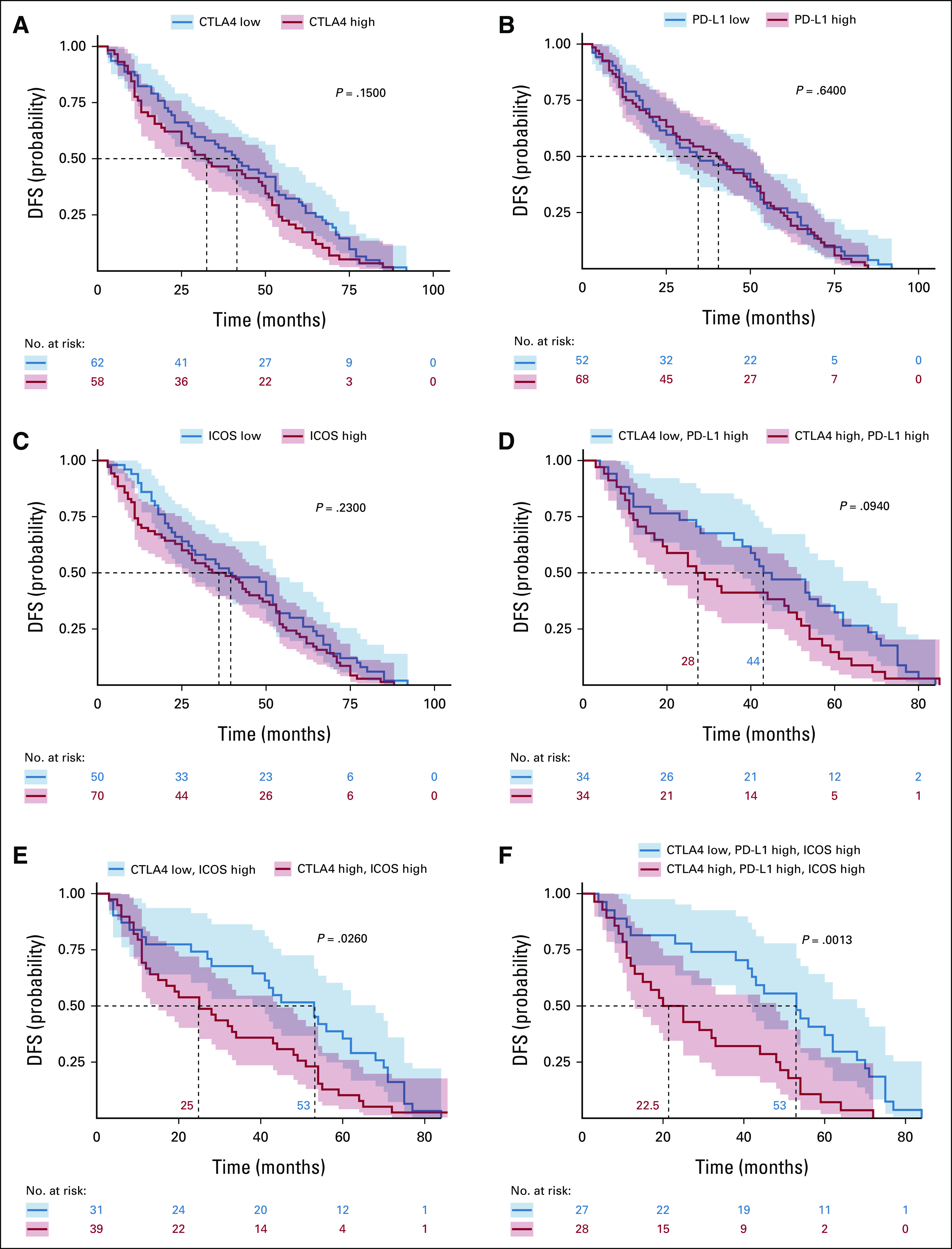
Immune checkpoints Kaplan-Meier DFS probability plots. DFS probability of patients classified on the basis of expression levels in normal tissue of (A) CTLA-4 alone (median survival of 58 and 62 months for the low and high groups, respectively); (B) PD-L1 alone (median survival of 34.5 and 40.5 months for the low and high groups, respectively); (C) ICOS alone (median survival of 39.5 and 36 months for the low and high groups, respectively); (D) coexpression of CTLA-4 and PD-L1 (median of 44 months for CTLA-4 low, PD-L1 high and 28 months for CTLA-4 high, PD-L1 high); (E) coexpression of CTLA-4 and ICOS (median of 53 months for CTLA-4 low, ICOS high and 25 months for CTLA-4 high, ICOS high); and (F) coexpression of CTLA-4, PD-L1, and ICOS (median of 53 months for CTLA-4 low, PD-L1 high, ICOS high and 22.5 months for CTLA-4 high, PD-L1 high, ICOS high). The table shown below the curves indicates the number of patients from each group who survived up to a given time (according to the values displayed in the *x*-axis in each figure) during the follow-up period (until 92 months when the recurrence of the last patient occured). Dotted lines point the median DFS in each caption. CTLA, cytotoxic T-cell lymphocyte; DFS, disease-free survival; PD-L1, programmed death-ligand 1.

Figure 3 shows the KM analysis incorporating the expression levels of *PD-L1*, *CTLA-4*, and *ICOS* alone or in combination. None of the three genes alone was significantly associated with DFS (Figs 3A and 3C).

DFS outcomes associated with *CTLA-4* low versus *CTLA-4* high status were not changed by the addition of high *PD-L1* expression (Fig 3D) but became statistically significant in the presence of high *ICOS* expression (Fig 3E). Indeed, the median survival was 53 months (for *CTLA-4* low, *ICOS* high in normal lung tissue compared with only 25 months for *CTLA-4* high, *ICOS* high; *P* = .026; Fig 3E).

Patients with *CTLA-4* high, *PD-L1* high, and *ICOS* high in normal lung tissue had the shortest median survival (22.5 months) that was significantly different from patients with *CTLA-4* low, *PD-L1* high, and *ICOS* high expression (53 months; *P* = .0013; Fig 3F).

### Combining DDPP Score and Normal Tissue Immune Status

**FIG 4.**
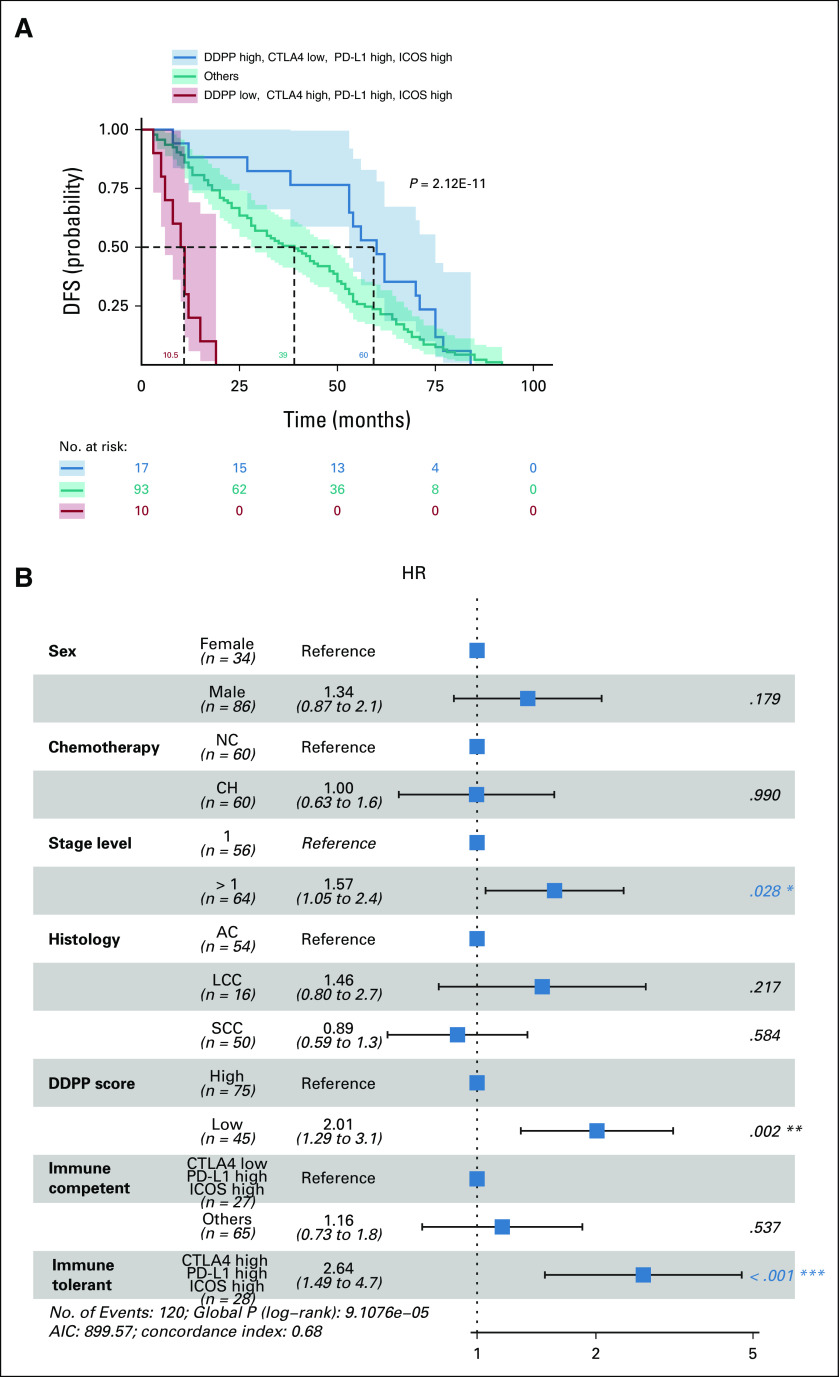
KM DFS probability plots and Forest plot analysis of combined IO and DDPP biomarkers. (A) KM plots of DFS probability of patients with low DDPP and immune-tolerant status (red line, median of 10.5 months) versus high DDPP and immune-competent status (blue line, median of 60 months) and all other combinations of DDPP and the three genes' levels (teal line, median of 39 months); (B) Forest plot of Cox proportional hazard model showing the HRs for various covariates (TNM stage, histology, DDPP, and immune status) included in the model. HR with > 1 (no-effect line) indicates a higher risk in disease recurrence. HR below 1 indicates a decrease in the risk for disease recurrence. *P* values with a 95% CIs are shown for each covariate included in the model. *,**,***, independent significant variables in multivariate analysis; AC, adenocarcinoma; AIC, akaike information criteria; CTLA, cytotoxic T-cell lymphocyte; DDPP, Digital Display Precision Predictor; DFS, disease-free survival; HR, hazard ratio; KM, Kaplan-Meier; LCC, large-cell carcinoma; NC, no-chemotherapy; PD-L1, programmed death-ligand 1; SCC, squamous cell carcinoma.

The KM DFS probability plots demonstrate that the highest correlation with the DFS is provided by the combined assessment of the three immune checkpoint genes in normal lung tissue and the DDPP score (*P* = 2.12E–11; Fig 4A).

A key determinant of outcome was the status of *CTLA-4*. Patients with low *CTLA-4* expression in the presence of high *PD-L1* and high *ICOS* (in normal tissue) and high DDPP score had the longest DFS compared with all others. The Forest plot of multivariate hazard Cox regression analyses (Fig 4B) shows that TNM, DDPP score, and immune status of normal lung tissues are independent prognostic markers.

## DISCUSSION

Lung cancer care is associated with poor outcome primarily because of the advanced stage of disease at the time of diagnosis and to limited effectiveness of currently available anticancer drugs. The TNM staging and the tumor histology constitute the current standard criteria for prognostication.^3-5^ Adjuvant chemotherapy after surgical resection has been shown to improve survival in patients with stage 2 or 3A disease, but its benefit in stage 1 patients has not been demonstrated, and its effectiveness is modest even in those with high risk of recurrence.^5,7^ New biomarkers are needed to identify those patients who are at high risk of tumor recurrence after surgery and might benefit from adjuvant treatment.

Our analysis of 120 patients with NSCLC is consistent with previous reports demonstrating the importance of TNM stage as a prognostic marker. However, the stage and histology alone could not predict the individual DFS variations observed or to predict the benefit of adjuvant therapy.

Using the DDPP methodology,^19^ we identified a transcriptomic biomarker signature and defined a DDPP low versus high score that significantly correlates with DFS. The cornerstone of the methodology was the exploration of transcription profiles of paired tumor and normal lung tissues from the same patient. This comparison allows distinguishing interpatient variability in gene expression in normal lung tissue from variability related to tumor transformation, thereby using each patient as his/her own control. Moreover, exploration of normal lung tissue enabled us to assess the status of the immune system in the host normal lung tissue in addition to tumor characteristics.

We found that for patients with NSCLC with stage 1 + 2 with DDPP low score, adjuvant chemotherapy was associated with improved DFS. However, the association between the use of adjuvant chemotherapy and DFS was not significant in the group of patients with DDPP high score, a more favorable risk group. These observations suggest that the DDPP score may be useful to identify patients with early-stage NSCLC who are at high risk of recurrence and could potentially benefit from adjuvant chemotherapy and also identify those with such a favorable prognosis that adjuvant chemotherapy is not likely to be beneficial.

The genes included in each of the six DDPP predictors are presented in detail in the Data Supplement and illustrate a significant enrichment in cancer-related biological pathways (control of transcription factors, control of cell cycle and proliferations, ubiquitin degradation of proteasome, and mitogen-activated protein kinase) indicating that ACC, SCC, and LCC are distinct diseases at the biological level. Pathway enrichment analysis using the MsigDB Hallmark 2020 database showed that the key pathway included in the DDPP predictor of the AC patients treated with surgery only was the G2-M checkpoint control (*P* = .0042 and adjusted *P* value for multiple comparisons = .012) while the key pathway for genes in the SCC predictor was IL6/JAK/STAT3 signaling (*P* = .0018, adjusted *P* = .015), and the key pathway in the LCC predictor was KRAS signaling pathway (adjusted *P* = .04). For patients treated with adjuvant chemotherapy, we found a significant enrichment of pathways involved in ion channel transporters and metabolism of nucleotides. The DDPP modeling, therefore, appears to yield results that are biologically coherent and relevant.

Our results also indicate that a higher coexpression of *CTLA-4*, *PD-L1*, and *ICOS* in normal lung tissues, presumed to induce an immune tolerance to tumor neoantigens, was associated with a shorter DFS after curative-intent surgery. Indeed, increased negative blockade of immune activation may limit the immune response to disseminated micrometastases.^20^ In contrast, an immune-competent profile would enable an efficient interception and elimination of circulating cells released during surgery and established micrometastases by activated immune cells.^21,22^ Finally, we explored the impact of combining DDPP and immune-tolerant profiling on the DFS and demonstrated that we could identify the patients with the highest risk of recurrence postsurgery, revealing a patient population with a clear need for further therapeutic intervention. In multivariate analysis, TNM stage, DDPP score, and immune-tolerant status of normal lung were found to be independent prognostic variables.

We recognize that our study has important limitations. First, the CHEMORES study, the source of biospecimens and clinical outcomes for our analysis, was not a prospective interventional trial and the decision of whether to administer postoperative adjuvant chemotherapy and the chemotherapy regimen administered was left to the treating physician. In the absence of random assignment, it is possible that there were important imbalances in prognostic factors between patients who did or did not receive adjuvant chemotherapy that are not accounted for in our analysis and could affect the validity of our findings. Second, we acknowledge that the numbers of patients with NSCLC with stage 1 + 2 are small and that the estimate of the effect size of adjuvant chemotherapy in the low-stage but high-risk (by DDPP score) patients is likely imprecise. Thus, our findings can be considered hypothesis-generating only and must be validated in a larger, prospective study.

Third, information about the tumor histological grade was not available for this cohort and is an established prognostic factor not included in our analyses.

The DDPP strategy to predict DFS for patients with resectable NSCLC requires further validation in a prospective study. A potential validation strategy would be to enroll patients with NSCLC undergoing surgery and collect tumor and normal tissues to analyze differential gene expression. For each patient, risk of recurrence will be determined but not provided to the treating physician. Patients will be followed until recurrence, and predicted versus observed recurrence rates will be analyzed.

If the prognostic utility of these analyses are validated, future prospective studies could examine the utility of adjuvant chemotherapy or chemoimmunotherapy in patients predicted to be at intermediate or high risk of recurrence, much as the TAILORx trial was used to validate the OncotypeDx recurrence score.^28^

## Data Availability

The data related to this paper have been submitted to the Array Express data repository at the European Bioinformatics Institute (http://www.ebi.ac.uk/arrayexpress/) under the accession numbers E-MTAB-1132 (Array express http://www.ebi.ac.uk/arrayexpress/) under the accession numbers E-MTAB-1132 (GE).^29^ For review process, all manuscript related data, related manuscript documents and the proprietary R codes are available at: https://drive.google.com/drive/folders/1sWYjRj86HyYrH5YGGWK-rFXRq_Jrs_4Y?usp=sharing.
